# Physicochemical Characterization and In Vitro Activity of Poly(ε-Caprolactone)/Mycophenolic Acid Amorphous Solid Dispersions

**DOI:** 10.3390/polym16081088

**Published:** 2024-04-13

**Authors:** Oroitz Sánchez-Aguinagalde, Eva Sanchez-Rexach, Yurena Polo, Aitor Larrañaga, Ainhoa Lejardi, Emilio Meaurio, Jose-Ramon Sarasua

**Affiliations:** 1 Department of Mining-Metallurgy Engineering and Materials Science, POLYMAT, Bilbao School of Engineering, University of the Basque Country (UPV/EHU), Plaza Ingeniero Torres Quevedo 1, 48013 Bilbao, Spain; oroitz.sanchez@ehu.eus (O.S.-A.); evagloria.sanchez@ehu.eus (E.S.-R.); aitor.larranagae@ehu.es (A.L.); emiliano.meaurio@ehu.eus (E.M.); jr.sarasua@ehu.es (J.-R.S.); 2 Polimerbio SL, Paseo Miramon 170, 20014 Donostia-San Sebastian, Spain; ypolo@polimerbio.com

**Keywords:** poly(ε-caprolactone) (PCL), mycophenolic acid (MPA), amorphous solid dispersions (ASDs), miscibility, interactions, drug release, cancer treatment

## Abstract

The obtention of amorphous solid dispersions (ASDs) of mycophenolic acid (MPA) in poly(ε-caprolactone) (PCL) is reported in this paper. An improvement in the bioavailability of the drug is possible thanks to the favorable specific interactions occurring in this system. Differential scanning calorimetry (DSC) was used to investigate the miscibility of PCL/MPA blends, measuring glass transition temperature (T_g_) and analyzing melting point depression to obtain a negative interaction parameter, which indicates the development of favorable inter-association interactions. Fourier transform infrared spectroscopy (FTIR) was used to analyze the specific interaction occurring in the blends. Drug release measurements showed that at least 70% of the drug was released by the third day in vitro in all compositions. Finally, preliminary in vitro cell culture experiments showed a decreased number of cancerous cells over the scaffolds containing MPA, presumably arising from the anti-cancer activity attributable to MPA.

## 1. Introduction

As new treatments and drugs appear for all kinds of diseases, we are also faced with great challenges to achieve a satisfactory application of these remedies. Although they may be effective in theory, most of the drugs that are being approved are not feasible in terms of their biopharmacological properties. The main causes are low permeability, poor solubility, or rapid elimination from the body. In fact, 90% of the drugs being developed are molecules with low water solubility, in addition to almost 40% of the drugs already approved [1,2,3]. The dimensions of this problem can be seen, for example, in the case of the oral administration of doses. In order to reach systemic circulation, the drug must be dissolved in the intestinal fluids of the gastrointestinal tract, which is difficult in the case of low solubility [4]. The cause of this low bioavailability is the different molecular arrangements, where the crystalline compounds are the ones that present the greatest problem [5]. In order to solve this problem, one of the established strategies is amorphization, which transforms low-energy crystalline substances into high-energy amorphous compounds, giving them greater solubility and bioavailability [6]. However, these amorphous solids are not thermodynamically stable because of their excess enthalpy, entropy, and free energies, which cause them to tend to form crystals [7]. For this reason, achieving the stability of these compounds is a great challenge.

One of the strategies used for this purpose is developing amorphous solid dispersions (ASDs). In the 1970s, Chiou and Riegelman defined the term solid dispersions as the dispersion of an active pharmaceutical ingredient (API) in an amorphous carrier in a solid state prepared by solvent, melting, or solvent-melting methods [7]. In these systems, there is a mixture at the molecular level between a polymer and the drug in an amorphous state, increasing its bioavailability [8,9,10,11,12]. It is known that the low thermodynamic stability due to the high energy of the amorphous state causes relaxation, nucleation, and recrystallization under different variables [13,14,15,16]. Thus, the role of the polymeric matrix is to inhibit this process and maintain the mixture in a single homogeneous phase [17,18]. To avoid this crystallization and maintain the mixture in the metastable region of the binary phase diagram, miscibility between the API and polymer is essential [19,20,21]. The kinetic stability provided by storage below the glass transition temperature (T_g_) must also be taken into account. In fact, according to Hancock et al., the stability of the mixture could be ensured for years by storing it at least 50 K below T_g_ [22,23]. One significant challenge in this system is the unpredictable nature of polymer–drug interactions [24].

**Scheme 1:**
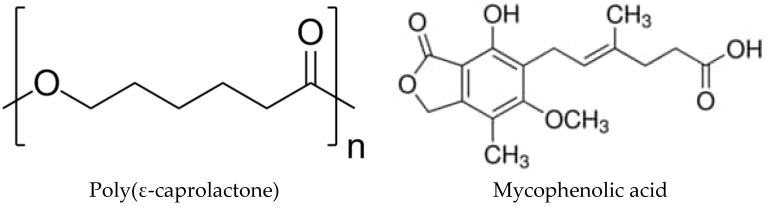
Chemical structures of PCL and MPA.

One interesting drug to test this system is mycophenolic acid (MPA—C_17_H_20_O_6_, 320 g/mol; aqueous solubility: 35.5 mg/L). Mycophenolic acid (Scheme 1) is an antibiotic produced by the *Penicillium* family and is best known for its use as an immunosuppressive agent to prevent rejection in organ transplants [25,26]. In addition, this drug has more biological properties, such as antifungal or antiviral properties [27]. It also has the potential to prevent and perhaps treat chronic allograft vasculopathy, as it can inhibit the proliferation of vascular smooth muscle cells (VSMCs), mesangial cells, and myofibroblasts [28]. However, one of the most striking properties is its ability to act against tumor cells of various types such as leukemia or lymphoma, among others [29]. This is because MPA is an inhibitor of inosine monophosphate dehydrogenase (IMPDH), which leads to the reduction of xanthine monophosphate (XMP), guanosine-5′-triphosphate (GTP), and deoxyguanosine triphosphate (dGTP), thus inhibiting the proliferation of lympholeukocytes and cancer cells [26,30]. Despite having so many favorable properties, the bioavailability of MPA in vivo is relatively poor due to the high clearance inside a living organism, which limits its possibility of clinical application [25]. This, in addition to its low aqueous solubility, makes it a perfect candidate for forming amorphous solid dispersions.

In this work, the polymer selected as the matrix to disperse MPA in amorphous form is poly(ε-caprolactone) (PCL), a biodegradable semicrystalline polyester. Its glass transition temperature is around −60 °C and its melting point at around 60 °C. The biodegradation of this polymer under physiological conditions has been reported to last several months to years [31,32], making it suitable for long-term biomedical applications. In this work, miscibility and interactions between PCL and MPA are studied to verify the suitability of this mixture for the formation of an amorphous solid dispersion. In addition, we separately tested the interaction of the blends containing increasing concentrations of MPA with both a non-cancerous fibroblast cell line (MRC5), approved by ISO 10993 for cytotoxicity studies [33], and a widely used immortalized HeLa cell line derived from cervical cancer [34].

## 2. Experimental Section

### 2.1. Starting Materials

Poly(ε-caprolactone) (PURASORB^®^ PC12 trade name) with an average molecular weight (M_w_) of 1.3 × 10^5^ g/mol and M_w_/M_n_ = 1.76 was purchased from Purac Biochem (Gorinchem, The Netherlands). Mycophenolic acid (C_17_H_20_O_6_, M = 320.34 g/mol) was obtained from Fluorochem Ltd. (Gossop, UK), and dichloromethane (DCM) was supplied by Labkem (Dublin, Ireland).

### 2.2. Blend Preparation

Films were prepared by solvent casting from dichloromethane (DCM) solutions containing 2.5 wt% of PCL/MPA blend at room temperature.

### 2.3. Differential Scanning Calorimetry (DSC)

A Modulated DSC Q200 from TA Instruments was used for thermal analyses. All the scans were performed in hermetic aluminum pans under nitrogen atmosphere with sample weights between 5 and 10 mg. Two scans from −80 °C to 160 °C with a scan rate of 20 °C/min were performed in order to measure glass transition temperatures (T_g_) in the second one.

### 2.4. Melting Point Depression Analysis

The melting point depression of MPA was observed in MPA-rich blends containing 0–20 wt% PCL. To obtain the melting temperature of MPA crystals, samples were heated in the DSC with a scan rate of 1 °C/min.

The samples were weighed again after the DSC scans, and no weight loss was observed during the thermal treatments.

### 2.5. Fourier Transform Infrared Spectroscopy (FTIR)

A Nicolet AVATAR 370 Fourier transform infrared spectrophotometer was used to record FTIR spectra of the blends, with a resolution of 2 cm^−1^ and averaged over 64 scans in the range of 400–4000 cm^−1^. Dichloromethane solutions containing 2 wt% of blends were cast on KBr pellets by evaporation of the solvent at room temperature. The absorbance of the samples was within the range where the Lambert–Beer law is obeyed.

### 2.6. In Vitro Drug Release

In vitro drug release experiments were performed for the PCL/MPA 99.95/0.05, 99.9/0.1, 99.8/0.2, 99.5/0.5, 99/1, and 98/2 blends. Round samples of PCL/MPA of Ø10 mm obtained by solvent casting were immersed in 1 mL of 0.1 M PBS buffer (pH 7.4) at 37 °C. At fixed intervals, samples of 200 μL were taken and replaced with fresh PBS at 37 °C. The drug concentration in solution was determined using a BioTech Sinergy H1M MicroPlate Reader (Minneapolis, MN, USA) using a calibration curve that was previously obtained measuring the absorbance at a wavelength of 305 nm for solutions of MPA in 0.1 M PBS.

The release kinetics of mycophenolic acid were examined by considering four mathematical models as follows:

(1) $$\mathrm{Zero}-\mathrm{order}:\;C_{t}/C_{\infty }=k_{0}t$$

(2) $$\mathrm{First}-\mathrm{order}:\;\ln\left(1-C_{t}/C_{\infty }\right)=-k_{1}t$$

(3) Higuchi: Ct/C∞=kht12

(4) $$\mathrm{Korsmeyer}–\mathrm{Peppas}:\;C_{t}/C_{\infty }=kt^{n}$$

where *C_t_* is the cumulative amount of the drug released at time *t*, *C*_∞_ is the starting amount of the drug, *n* is the release exponent, and *k*_0_, *k*_1_, *k_h_*, and *k* are the kinetic constants. Zero-order kinetics (Equation (1)) represents a release process that is controlled by the relaxation of polymeric chains, independent of its concentration and with a constant release rate. The first-order kinetics (Equation (2)) model represents a drug release rate that depends on its concentration [32]. Higuchi (Equation (3)) describes drug release as a diffusion process based on Fick’s law, square root time-dependent. If the release mechanism is not well known or when more than one type of release phenomena could be involved, the Korsmeyer–Peppas (Equation (4)) model is applied. It is possible to define whether the release happens by Fickian diffusion, anomalous transport, Case-II transport, or Super Case-II transport depending on the values obtained for the release exponent, *n* [35,36].

### 2.7. In Vitro Cell Culture Experiments

In vitro cell culture experiments were performed on the PCL/MPA 99.5/0.5, 99/1, and 98/2 blends. Circular samples of PCL/MPA of Ø6 mm were obtained, and each side was sterilized for 30 min under UV light. Either the immortalized HeLa cell line (ATCC, Manassas, VA, USA) derived from cervical cancer or the non-cancerous fibroblasts MRC5 (CCL-171, ATCC, Manassas, VA, USA) derived from lung tissue were drop-seeded over the materials at a concentration of 25,000 cells per scaffold. After 1 h, 480 mL of prewarmed DMEM (Fisher Scientific, Madrid, Spain) at 37 °C supplemented with 10% fetal bovine serum (FBS) (Fisher Scientific, Madrid, Spain), 1% L-glutamine (Fisher Scientific, Madrid, Spain), and penicillin/streptomycin (Fisher Scientific, Madrid, Spain) were added. PCL films were used as the negative control, and for the positive control, MPA in dissolution at a concentration of 300 ppm was dissolved on the culture media and filtrated (0.2 µm). Cells were incubated at 37 °C and 5% CO_2_ in a standard cell culture incubator.

### 2.8. Immunostaining

After 1 or 3 days in vitro (DIV), samples were fixed with 4% paraformaldehyde (PFA) (Fisher Scientific, Spain) and permeabilized with 0.3% triton-X100 (Fisher Scientific, Spain) in PBS (Fisher Scientific, Spain) containing 1% Bovine Serum Albumin (BSA) (Sigma Aldrich, Spain). For the staining, rhodamine/phalloidin (Fisher Scientific, Madrid, Spain) and DAPI, 4′,6-diamidino-2-phenylindole dihydrochloride (Fisher Scientific, Madrid, Spain) were diluted in 1% PBS BSA and incubated for 1.5 h. After washing each sample 2 times in PBS containing 0.1% Tween-20 (Fisher Scientific, Madrid, Spain) and 1 time in PBS, the samples were mounted using mounting medium (Abcam, Waltham, MA, USA). The samples were analyzed in an inverted fluorescence microscope (Nikon Eclipse Ts2). For cell quantification studies, 5 different points were taken.

### 2.9. Cell Count and Statistical Analysis

For cell counts, five aleatory images of 0.1 mm^2^ were taken for each of the triplicates in each condition, and nuclear DAPI labeling was used to calculate the total number of cells. The data were subjected to one-way analysis of variance (ANOVA) using Kruskal–Wallis followed by Dunn’s post hoc test. The level of significance was set at *p* < 0.05. The results were presented as mean ± SD or SEM.

## 3. Results and Discussion

### 3.1. Miscibility Analysis by Differential Scanning Calorimetry (DSC)

When two components are miscible, a single glass transition temperature (T_g_) between the T_g_ of each material, which changes progressively with the composition, is expected [37,38]. On the contrary, the detection of more than one single value would indicate a separation into individual amorphous phases within the system. Different methods have been employed to predict the glass transition temperature of amorphous binary systems, such as the Gordon–Taylor (GT), Couchman–Karasz (CK), and Fox equations (Equation (5)). Considering that the Fox equation was developed to analyze systems formed by components of equal densities, it is appropriate to use it to estimate this intermediate T_g_, as the densities of PCL and MPA are 1.14 g/cm^3^ and 1.3 g/cm^3^, respectively [39]:

(5) $$\frac{1}{T_{gb}}=\frac{w_{1}}{T_{g1}}+\frac{w_{2}}{T_{g2}}$$

where *w*_1_ and *w*_2_ are the weight fractions of components 1 and 2, respectively, *T_g_*_1_ and *T_g_*_2_ are the glass transition temperatures of the pure components, and *T_gb_* is the glass transition temperature of the blend.

**Figure 1:**
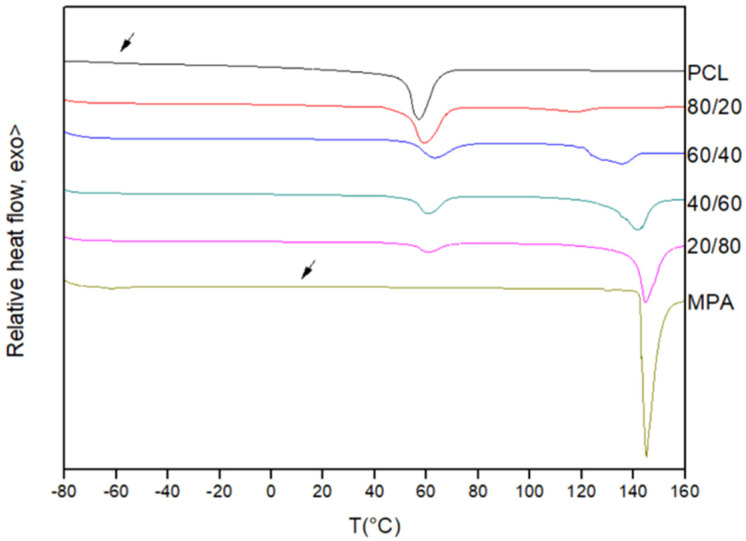
First scan DSC traces for PCL, MPA, and PCL/MPA blends.

**Figure 2:**
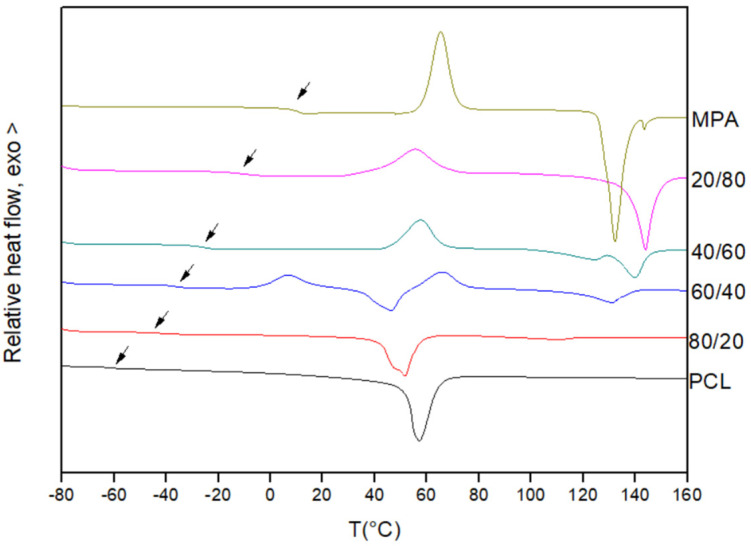
Second scan DSC traces for PCL, MPA, and PCL/MPA blends.

Figure 1 shows the first scan DSC traces obtained for the pure components and for different PCL/MPA blends. As can be seen, pure PCL is a semicrystalline polymer displaying a glass transition temperature located at about −60 °C and a melting endotherm at about 60 °C. On the other hand, MPA is a crystalline compound melting at 145 °C, which can be also supercooled to undergo a glass transition at 11 °C after reheating the quenched melt (see Figure 2).

**Figure 3:**
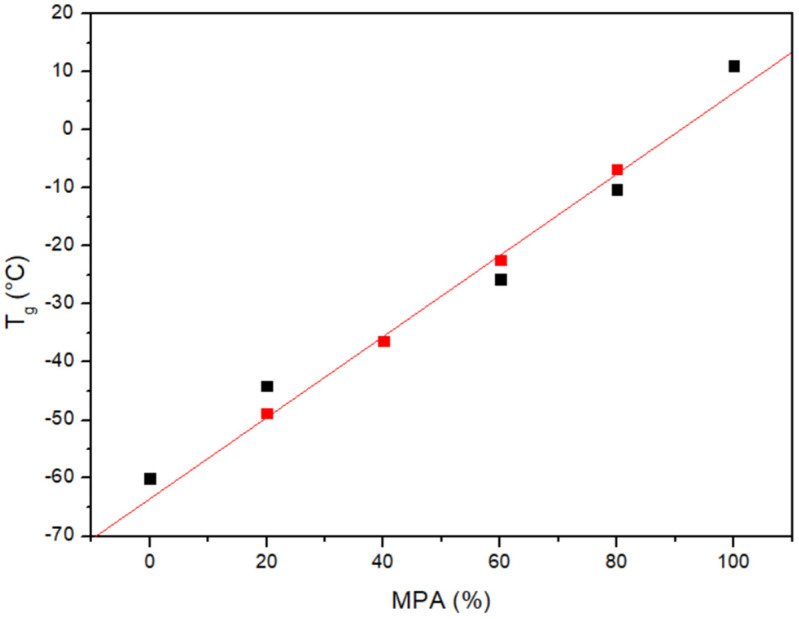
Glass transition temperature versus composition for the PCL/MPA system: (■) experimental values and (■) Fox equation.

As can be seen in Figure 2, the PCL/MPA blends show composition-dependent single glass transitions located close to the values predicted using the Fox equation (see Table 1 and Figure 3). Consequently, it can be concluded that the two components are completely miscible in the amorphous phase. Furthermore, the melting temperature of PCL decreases as the content of MPA increases. Furthermore, the crystallization of PCL is totally suppressed when the drug composition exceeds 50 wt%.

### 3.2. Melting Point Depression Analysis

If the free energy of the mixing of the two components (ΔG_mix_) is negative, a system can be considered thermodynamically miscible.

(6) $$∆G_{mix}=∆H_{mix}-T∆S_{mix}$$

where ΔH_mix_ and ΔS_mix_ are the enthalpy and entropy of mixing, respectively. TΔS_mix_ is always positive since the entropy of mixing is added to the entropy of melting, making the entropy change in a miscible blend larger than in the pure component. Consequently, the sign of ΔG_mix_ depends on the value of ΔH_mix_. In order to avoid phase separation, the cohesive interactions need to be lower than the sum of adhesive interactions, generating a favorable enthalpy of mixing. The miscibility between two components in terms of the change in the Gibbs free energy can be described using the melting point depression method, based on Flory–Huggins theory. According to this method, the melting point temperature of the drug will decrease as the polymer content in the mixture increases if the cohesive forces in the pure components are weaker than the adhesive forces between the drug and the polymer [32,40]. Flory’s relationship can be used to analyze the depression of the equilibrium melting point:

(7) $$\frac{1}{T_{m}}-\frac{1}{T_{m}^{0}}=\frac{-R}{∆H_{2u}}\frac{V_{2u}}{V_{1u}}\left(\frac{\ln\phi_{2}}{m_{2}}+\left(\frac{1}{m_{2}}-\frac{1}{m_{1}}\right)\phi_{1}+\chi_{12}\phi_{1}^{2}\right)$$

where $T_{m}^{0}$ is the equilibrium melting point of the pure crystallizable component and $T_{m}$ is the equilibrium melting point of its blends; the subscripts 1 and 2 refer to the amorphous and crystallizable components, respectively. R is the universal gas constant, while $∆H_{2u}$ is the heat of fusion per mole of crystalline repeat units. $V_{u}$ is the molar volume of the repeating unit, m is the degree of polymerization, ϕ is the volumen fraction, and $\chi_{12}$ is the interaction parameter.

In order to apply Equation (7), the molar volume of MPA ($V_{2}=246.3$ cm^3^/mol) can be considered as the molar volume of the lattice sites, resulting in $m_{2}=1$. The same volume can be taken as the molar volume of the polymeric repeat unit $V_{2}=V_{1u}$. Since $m_{1}=V_{pol}/V_{1u}$ is large, $1/m_{1}\approx 0$. As a result, Equation (7) simplifies to:

(8) $$\frac{1}{T_{m}}-\frac{1}{T_{m}^{0}}=\frac{-R}{∆H_{2}}\left(\ln\phi_{2}+\phi_{1}+\chi \phi_{1}^{2}\right)$$

**Figure 4:**
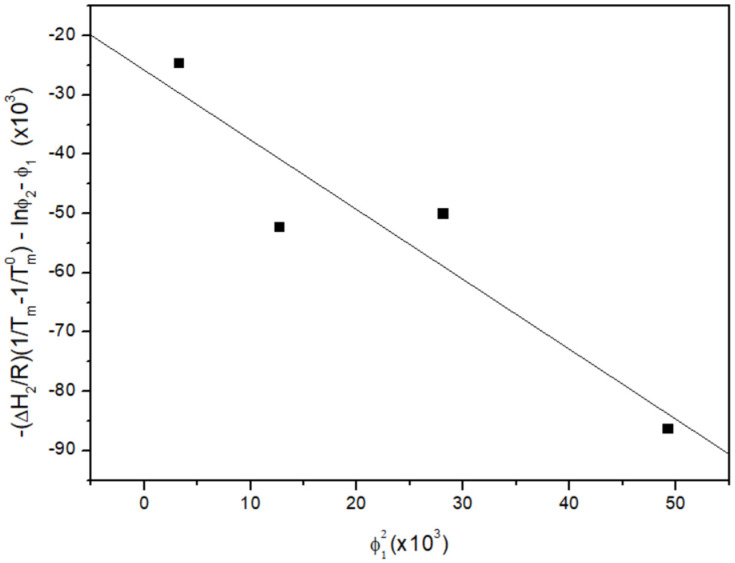
Analysis of the melting temperature of MPA according to Equation (8) for the PCL/MPA system. The slope of the plot gives the interaction parameter χ = −1.18.

The melting points of pure components and different PCL/MPA blends were measured at a low heating rate (1 °C/min). The average melting point of pure MPA is $T_{m}^{0}=140.3$ °C, and this temperature is decreased by nearly 5 °C when 20 wt% PCL is added to the blend. The data obtained for each blend can be seen in Table 2. These results, with the average melting enthalpy of pure MPA ($∆H_{MPA}=114.7$ J/g) were used to plot Equation (8) as a function of the square of the volume fraction of the polymer, $\phi_{1}^{2}$. The slope of this plot, which can be seen in Figure 4, gives an approximation of the interaction parameter of χ=−1.18. The negative values for the interaction parameter indicate an exothermic reaction, confirming a thermodynamically miscible blend. It is also possible to calculate the interaction energy density, B, at the melting temperature of MPA according to Equation (9):

(9) $$\chi =\frac{BV_{r}}{RT}$$

where $V_{r}$ is a reference volumen ($V_{r}=V_{2}=246.3$ cm^3^/mol), yielding B=−16.5 J/cm^3^.

### 3.3. Fourier Transform Infrared Spectroscopy (FTIR)

**Figure 5:**
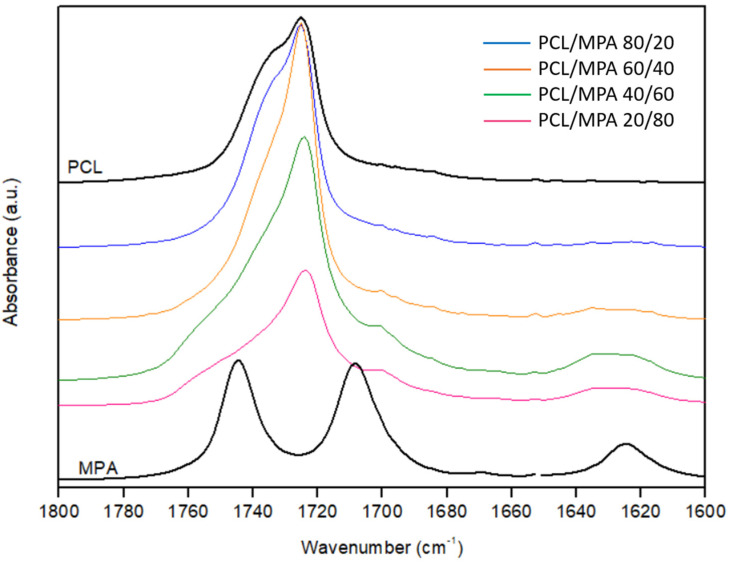
Carbonyl stretching region for pure PCL and MPA and PCL/MPA blends of different compositions.

**Figure 6:**
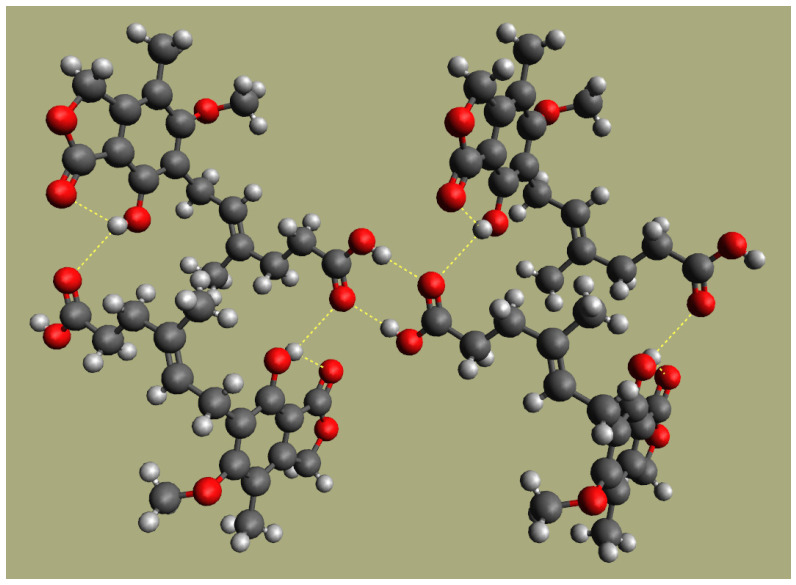
Hydrogen bonding in crystalline MPA (see text).

The analysis of the changes observed in the infrared spectrum upon blending provides information about the changes in specific interactions and can eventually aid in explaining the energetic contributions driving the miscibility of the system. In the PCL/MPA system, both the carbonyl and the hydroxyl stretching regions are of main interest because hydrogen bonding interactions can be expected for those groups. Figure 5 shows the carbonyl stretching region for PCL, MPA, and their blends. The spectrum of pure PCL shows a peak at 1725 cm^−1^ attributable to crystalline PCL and a shoulder at 1735 cm^−1^ arising from the amorphous phase [32,40]. On the other hand, pure MPA shows two different peaks located at 1744 and 1708 cm^−1^ attributable, respectively, to the lactone carbonyl and the carboxylic acid carbonyl. Both locations are at the lower end of the spectral ranges corresponding to those functional groups [41] because of the hydrogen bonding interactions occurring in pure MPA. Figure 6 sketches these interactions as derived from XRD studies [42,43,44]. As it can be seen, in pure MPA, the molecules are joined in the crystal by carboxylic acid groups forming dimers, along with bifurcated hydrogen bonds between the hydroxyl group and the carboxylic acid carbonyl (absorption band at 1708 cm^−1^). In addition, an intramolecular bifurcated hydrogen bond red shifts the absorption of the lactone carbonyl to the reported wavenumber (1744 cm^−1^).

The PCL/MPA 20/80 and 40/60 blends show a major peak located at about 1724 cm^−1^, accompanied by two shoulders at higher wavenumbers located at about 1735 cm^−1^ and 1750 cm^−1^. At these compositions, PCL is almost in amorphous form according to the DSC results (hence, the contribution corresponding to crystalline PCL should be negligible), and the absorption bands corresponding to MPA are expected to prevail over those of PCL; hence, the band at 1724 cm^−1^ is most likely attributable carboxylic acid carbonyls forming dimers in the amorphous phase. This band is probably strongly overlapped with PCL carbonyls hydrogen bonded with hydroxyl groups present in MPA, but unfortunately, these two components are not distinguishable. The shoulder at about 1735 cm^−1^ can be attributed to free C=O groups in PCL and the one at about 1750 cm^−1^ to lactone carbonyls in the amorphous phase.

**Figure 7:**
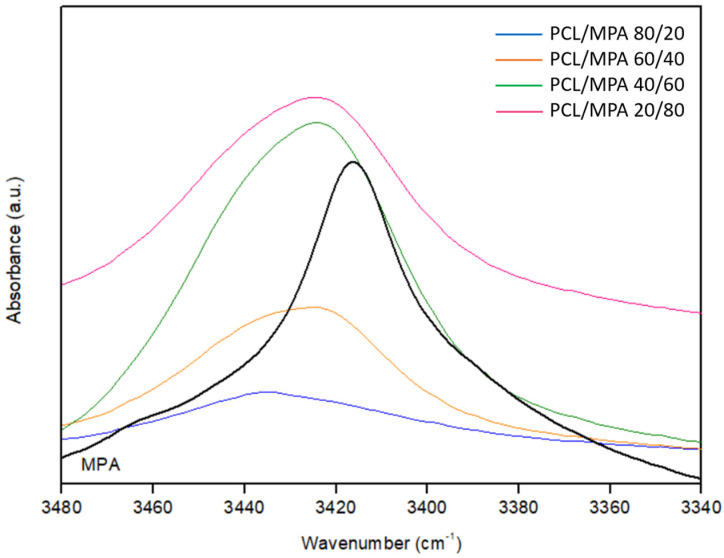
Hydroxyl stretching region for pure MPA and PCL/MPA blends of different compositions.

Finally, Figure 7 shows the hydroxyl stretching region for MPA and its blends with PCL. As can be seen, the OH stretching band in pure MPA is located at about 3416 cm^−1^, and blending broadens the band and shifts it to higher wavenumbers. Band broadening is a consequence of the presence of amorphous MPA, while shifting to higher wavenumbers can be attributed to weaker hydrogen bonding interactions in the blends compared with pure MPA. Despite the weaker nature of the interactions, the energetic balance will still render favorable to miscibility as long as the blend achieves a larger number of interactions, arising from the introduction of additional interacting groups (the PCL carbonyls).

### 3.4. In Vitro Drug Release

**Figure 8:**
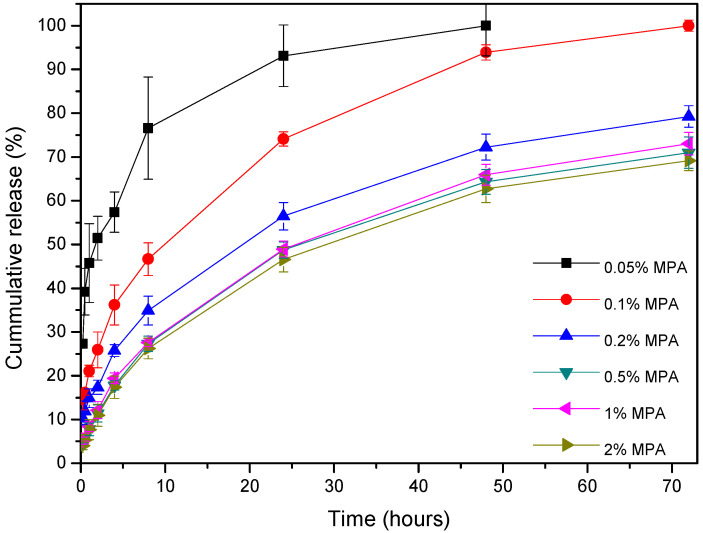
Drug release profiles of PCL/MPA films containing different drug concentrations immersed in 0.1 PBS buffer at 37 °C, shown in %.

**Figure 9:**
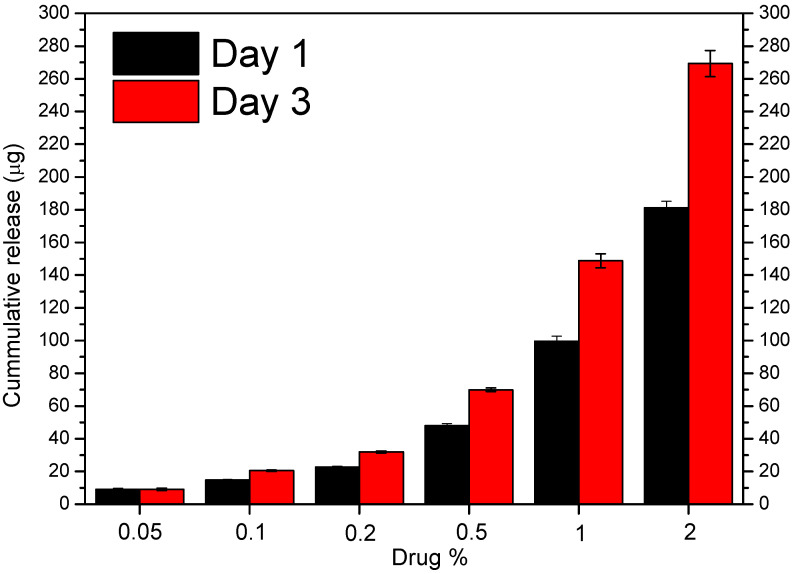
Drug release profiles of PCL/MPA films containing different drug concentrations immersed in 0.1 PBS buffer at 37 °C, shown in μg.

Figure 8 shows the in vitro release profiles of MPA from different PCL/MPA blend (0.05, 0.1, 0.2, 0.5, 1, and 2 wt% MPA) samples for 3 days (72 h). From the first moment, the release of MPA starts, which is faster in the samples with the lowest drug content. By the third day, the samples of 0.05 and 0.1 wt% MPA had released all the drugs, while the rest of the samples had released between 69 and 79 wt% of the total amount. Figure 9 shows that the release rate slowed down after the third day following an asymptotic tendency; thus, it is thought that the remaining drug will be fully released when bulk erosion begins.

The amorphous MPA dissolved in the matrix can easily travel across the polymer matrix reaching the outer solution as the solution temperature (37 °C) is higher than the glass transition temperature of PCL (−60 °C), allowing the chains to have enough mobility.

According to the results observed in Table 3, the release mechanisms vary depending on the concentration of MPA. For those with the lowest concentration (0.05 and 0.1 wt% MPA), the model that fits the results is first-order [45], while for the rest of the concentrations, a trend toward the Higuchi model is seen [46]. Therefore, at very low concentrations, the release kinetics depends on the concentration, while as the amount of MPA increases, the drug is released by a diffusion process based on Fick’s law, square root time-dependent.

### 3.5. Cell Viability with HeLa Immortalized Cancer Cells

**Figure 10:**
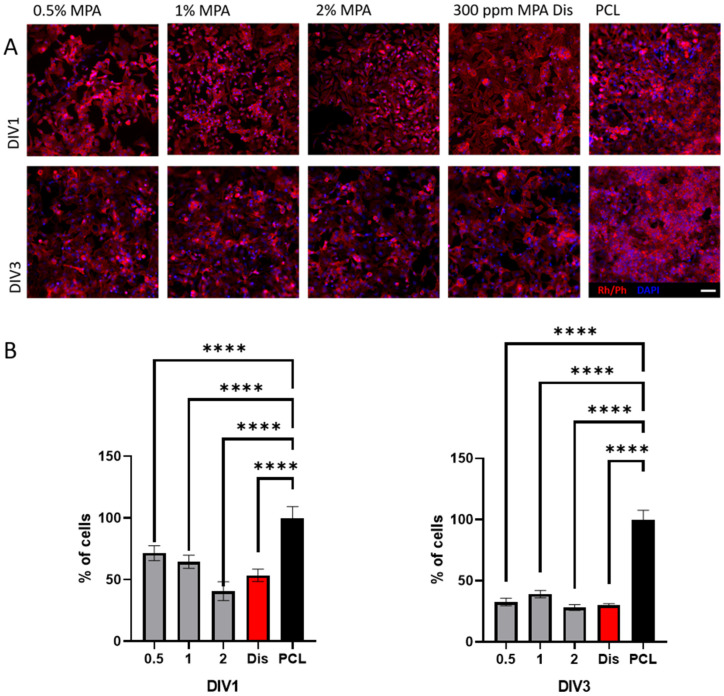
(**A**) Immunofluorescence assays against rhodamine/phalloidin (Rh/Ph) in red sowing the cytoskeleton and DAPI in blue showing the nuclei of HeLa cells cultured over PCL/MPA scaffolds containing increasing amounts of MPA. As a negative control, HeLa cells were cultured over PCL scaffolds, and as a positive control, HeLa cells cultured over PCL scaffolds were incubated with 300 ppm MPA in solution. (**B**) Quantification of the percentage of cells over the scaffolds at DIV1 and DIV3. (**** *p* < 0.0001 compared to the PCL scaffold at the same time points. Dunn’s or Holm–Sidak method one-way ANOVA analysis of variance on ranks.) Scale bar 50 μm.

In the last decade, some groups have reported the suppression of proliferation and the enhancement of apoptosis mediated by MPA in cancerous cells [26,47,48]. These properties are attributed to several interactions of the MPA with molecules involved in the cell cycle, cell death, cell proliferation, and movement [49]. Here, the combination of PCL with MPA as a possible anti-cancer therapy was studied. For this purpose, HeLa cells, a widely used immortalized cell line derived from cervical cancer were chosen. First, the attachment and proliferation capabilities of HeLa cells were analyzed on PCL scaffolds containing increasing concentrations of MPA after 1 and 3 days post-seeding (DIV1 and DIV3 respectively) by immunofluorescence assays against DAPI and rhodamine/Phalloidin (Rh/Ph). Results at DIV1 suggested that the PCL/MPA substrates affected the number of attached HeLa cells in a dose-dependent manner, achieving even the same impairment on cell viability as the MPA in solution (Dis) (PCL/MPA 0.5 71.5 ± 6.1%; PCL/MPA 1 64.6 ± 5.3%; PCL/MPA 2 40.1 ± 7.6%; Dis 53.6 ± 5%; compared with the control of PCL 100 ± 9.2%; *p* < 0.0001, one-way ANOVA). The results were further demonstrated at DIV3, when again, the number of the HeLa cells over the scaffolds containing MPA was reduced with respect to the PCL control (PCL/MPA 0.5 32.6 ± 3.1%; PCL/MPA 1 39.1 ± 5.3%; PCL/MPA 2 28.2 ± 2.4%; Dis 30.0 ± 1.4%; PCL 100 ± 7.6%; *p* < 0.0001, one-way ANOVA) (Figure 10). The results are in accordance with other studies where MPA caused the impairment of HeLa cell viability [49,50]. But the clearance effect of the tissues in vivo [51] must also be taken into consideration, where the slow release of MPA by the scaffolds may be an advantage compared with the direct drug administration [52]. Moreover, in future experiments, these scaffolds could be further engineered to adjust the release of the MPA to the kinetics of the drug by just modifying the degradation ratio of the scaffolds or the anchoring mechanism of the drug [52], which is a promising tool for anti-cancer drug delivery.

### 3.6. Cell Viability with Fibroblasts

**Figure 11:**
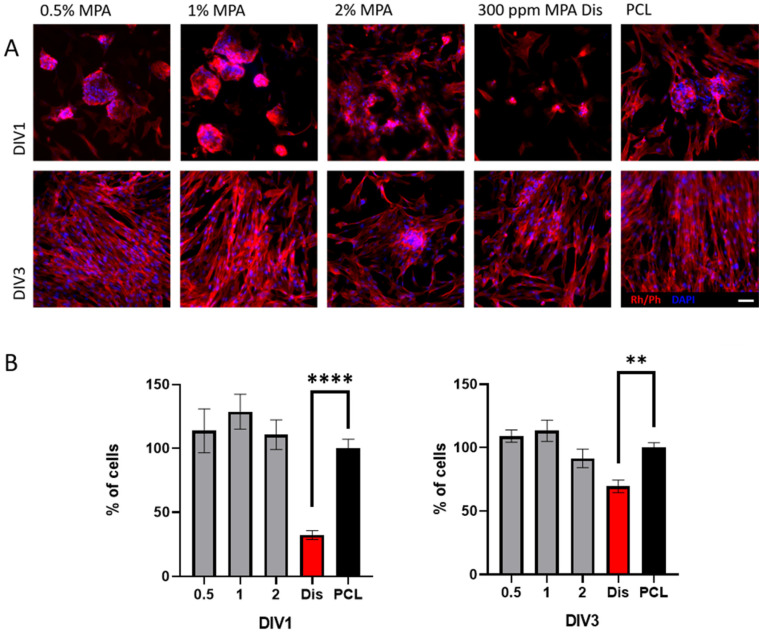
(**A**) Immunofluorescence assays against rhodamine/phalloidin (Rh/Ph) in red showing the cytoskeleton and DAPI in blue showing the nuclei of MRC5 cells cultured over PCL/MPA scaffolds containing increasing amounts of MPA. As a negative control, MRC5 cells were cultured over the PCL scaffolds, and as a positive control, MRC5 cells cultured over PCL scaffolds were incubated with 300 ppm MPA in dissolution. (**B**) Quantification of the percentage of cells over the scaffolds at DIV1 and DIV3. (** *p* < 0.05 and **** *p* < 0.001 compared to the PCL scaffold at the same time points. Dunn’s or Holm–Sidak method one-way ANOVA analysis of variance on ranks.) Scale bar 50 μm.

One big hallmark of anti-cancer therapies is the possibility of treating cancer cells without affecting non-cancerous cells [53]. In this regard, the adhesion and proliferation of the non-cancerous fibroblast cell line MRC5 or CCL-171 were tested. This cell line is not immortalized, and according to ISO 10993-5:2009, it is considered a good control for cytotoxic experiments [33]. The results suggested that the number of MRC5 cells able to attach and proliferate on the PCL scaffolds containing increasing amounts of MPA at DIV1 (PCL/MPA 0.5 113.7 ± 17.2%; PCL/MPA 1 128.7 ± 13.7%; PCL/MPA 2 110.7 ± 11.6%; PCL 100 ± 7.1%) and DIV3 (PCL/MPA 0.5 109.1 ± 4.8%; PCL/MPA 1 113.3 ± 8.4%; PCL/MPA 2 91.6 ± 7.3%; PCL 100 ± 3. %) were similar to the pristine PCL scaffolds (Figure 11). Surprisingly, both at DIV1 (Dis 32.2 ± 3.5%; *p* < 0.05, one-way ANOVA) and DIV3 (Dis 69.4 ± 4.9%; *p* < 0.05, one-way ANOVA), the MRC5 cells seeded on the PCL scaffolds together with MPA in solution appeared to have an impaired proliferation. In this regard, several tumorigenic and non-tumorigenic cell lines showed resistance to MPA treatment by converting MPA into its inactive form 7-*O*-glucoronide [54,55,56], which might explain the results obtained with the scaffolds. However, further research is needed to study the anti-cancer capabilities of the MPA blended in PCL films with other cancerous and non-cancerous cell lines and the advantages and disadvantages compared with MPA in solution.

## 4. Conclusions

In the present work, the possibility of forming an amorphous solid dispersion of mycophenolic acid using poly(ε-caprolactone) as a matrix was confirmed. Miscibility between the two compounds was observed in thermal properties. On the one hand, the intermediate glass transition temperature criterion was confirmed for all the compositions. On the other hand, the analysis of the melting point depression of the MPA crystals resulted in a negative interaction parameter, χ=−1.18, indicating favorable interactions between the polymer and the bioactive molecule.

The analysis by FTIR spectroscopy for the PCL/MPA blends does not allow us to clearly confirm the occurrence of hydrogen bonding interactions between the hydroxyl groups present in MPA and the C=O groups of PCL because of the complex nature of the C=O stretching region. Nevertheless, the overall results observed in both the C=O and O-H stretching regions follow the typical trends observed in other polymer blends with miscibility driven by hydrogen bonding interactions.

The potential application of this PCL/MPA amorphous solid dispersion as drug delivery matrices was proven, as at least 70% of the drug was delivered by the third day in vitro in all compositions. In addition, in vitro cell culture experiments with HeLa and MRC5 cells showed that it is possible to maintain the active form of MPA for cancer treatment, as there is a decreased viability of cancerous cells when cultured over PCL materials containing MPA. Here, the potential beneficial outcome of dissolving MPA in PCL matrices for anti-cancer treatment was observed, although further research is needed to study the anti-cancer capabilities with other cell lines and the mechanism, advantages, and disadvantages these amorphous solid dispersions offer compared with crystalline MPA.

## Figures and Tables

**Table 1:** Thermal properties of PCL/MPA blends.

PCL/MPA	T_g_ Experimental (°C)	T_g_ Theoretical (Fox) (°C)	T_m_ PCL (°C)	ΔH_f_ PCL (J/g)
**PCL**	−60.0	-	57.2	66.4
**80/20**	−44.1	−48.8	51.7	49.8
**60/40**	−36.3	−36.3	46.4	25.9
**40/60**	−25.7	−22.4	-	-
**20/80**	−10.2	−6.8	-	-
**MPA**	11.1	-	-	-

**Table 2:** Melting temperatures of MPA obtained from 1 °C min^−1^ scan rates.

MPA wt%	T_m_ (°C)		
	Sample 1	Sample 2	Sample 3
**100**	139.2	140.5	140.9
**95**	139.6	139.9	138.3
**90**	138.4	138.1	137.4
**85**	137.4	138.9	136.8
**80**	135.8	135.7	135.9

**Table 3:** Fitting of the release data to the mathematical models for drug release kinetics. R^2^ is the correlation coefficient and n is the release exponent.

MPA %	Zero-Order	First-Order	Higuchi	Korsmeyer–Peppas	
**0.05**	R^2^ = 0.64	R^2^ = 0.94	R^2^ = 0.87	R^2^ = 0.81	n = 0.58
**0.1**	R^2^ = 0.86	R^2^ = 0.99	R^2^ = 0.98	R^2^ = 0.92	n = 0.5
**0.2**	R^2^ = 0.74	R^2^ = 0.88	R^2^ = 0.94	R^2^ = 0.95	n = 0.46
**0.5**	R^2^ = 0.75	R^2^ = 0.85	R^2^ = 0.94	R^2^ = 0.99	n = 0.5
**1**	R^2^ = 0.77	R^2^ = 0.89	R^2^ = 0.95	R^2^ = 0.99	n = 0.5
**2**	R^2^ = 0.77	R^2^ = 0.87	R^2^ = 0.95	R^2^ = 0.99	n = 0.5

## Data Availability

The data presented in this study are available on request from the corresponding author.
